# Lysine lactylation analysis of proteins in the heart of the Kawasaki disease mouse model

**DOI:** 10.3389/fcell.2025.1550220

**Published:** 2025-03-06

**Authors:** Wenyu Zhuo, Mingyang Zhang, Jiajia Tan, Yang Gao, Yan Wang, Nana Wang, Jin Ma, Jiaying Zhang, Zhiheng Liu, Haitao Lv, Ying Liu

**Affiliations:** ^1^ Institute of Pediatric Research, Children’s Hospital of Soochow University, Suzhou, Jiangsu, China; ^2^ Department of Cardiology, Children’s Hospital of Soochow University, Suzhou, Jiangsu, China; ^3^ Department of Pediatrics, The First People’s Hospital of Lianyungang, Xuzhou Medical University Affiliated Hospital of Lianyungang (Lianyungang Clinical College of Nanjing Medical University), Lianyungang, China; ^4^ Department of Cardiology, The Affiliated Xuzhou Children’s Hospital of Xuzhou Medical University, Xuzhou, Jiangsu, China; ^5^ Department of Pharmacy, Children’s Hospital of Soochow University, Suzhou, Jiangsu, China

**Keywords:** Kawasaki disease, lysine lactylation, proteomic analysis, bioinformatics analysis, mouse model

## Abstract

**Introduction:**

Kawasaki disease (KD) is a medium-vessel vasculitis predominantly affecting children under 5 years of age and may involve the coronary arteries.

**Methods:**

A mouse KD model was induced by *Candida albicans* cell wall extracts (CAWS), cardiac tissues were analyzed through integrated lactylomic and proteomic profiling. The lysine lactylation (Kla) results were normalized to the proteomic data.

**Results:**

Elevated serum lactate and lactate dehydrogenase (LDH) levels were observed in KD patients. Given lactate’s role as a substrate for Kla, this study investigated Kla modifications in KD. Proteomic analysis identified 150 upregulated proteins and 18 downregulated proteins, with 38.1% located in the cytoplasm and significant enrichment in immune-related pathways. After normalization, 41 sites in 37 proteins were found to be upregulated in the Kla data, with no downregulated sites. Approximately 67.57% of the altered proteins were localized in the mitochondria. Bioinformatics analysis indicated alterations in aerobic respiration, energy production and conversion, and key immune- and metabolism-related pathways.

**Discussion:**

This study enhances the understanding of Kla modifications in the development of KD and may inform targeted therapies for its prevention and improved prognosis.

## Introduction

Kawasaki disease (KD) is a medium-vessel vasculitis that primarily affects children less than 5 years of age (Burns and Glodé, 2004). If left untreated within the initial 10 days of disease onset, preferably within the first 7 days, it can lead to development of coronary artery aneurysms. KD initially presents as necrotizing arteritis with neutrophilic infiltration and is characterized by a fever lasting at least 5 days. Subsequently, subacute and chronic changes occur, accompanied by the proliferation of luminal myofibroblasts, which may cause arterial stenosis. Once KD is diagnosed, echocardiography should be promptly performed, followed by follow-up echocardiograms at 1–2 weeks and 4–6 weeks (Kimura et al., 2017; Wang et al., 2021; Newburger, 2017).

Although KD was first reported by Dr. Kawasaki in 1967 (Kawasaki et al., 1974) and medical research has advanced over the years, its incidence continues to increase rapidly, making it the primary cause of acquired heart disease in developed countries (McCrindle et al., 2017). Further exploration of the molecular changes during the progression of KD is necessary to enhance the understanding of the disease. Due to the difficulty in obtaining coronary artery tissue from KD patients, a mouse model induced by injecting *Candida albicans* cell wall extracts (CAWS) is widely used (Ohno, 2004). Based on the disease course of KD patients, the sub-acute stage—suitable for investigating coronary artery lesions—is considered to occur 14 and 28 days after the final CAWS injection, which is .

Lysine lactylation (Kla), a recently discovered post-translational modification (Zhang et al., 2019), is involved in multiple cellular activities. Kla requires lactate, a glycolysis metabolite regulated by lactate dehydrogenase (LDH) (Xia et al., 2024). Kla has attracted attention in the cardiovascular and inflammatory research. For example, the lactylation of PKM2 can suppress inflammatory metabolic adaptation in pro-inflammatory macrophages (Wang et al., 2022), YY1 lactylation in microglia promotes angiogenesis (Wang et al., 2023), and α-myosin heavy-chain lactylation helps maintain the sarcomeric structure and alleviates heart failure (Zhang et al., 2023). Therefore, it was hypothesized that Kla might play a role in the progression of KD.

In this study, healthy children (normal control, NC), children with fever, and KD patients were recruited to measure lactate and LDH levels in their peripheral blood. Additionally, hearts from KD mouse models were collected for lactylation proteomics analysis to explore the lactylome of KD hearts.

## Materials and methods

### Study cohort and sample collection

**FIGURE 1:**
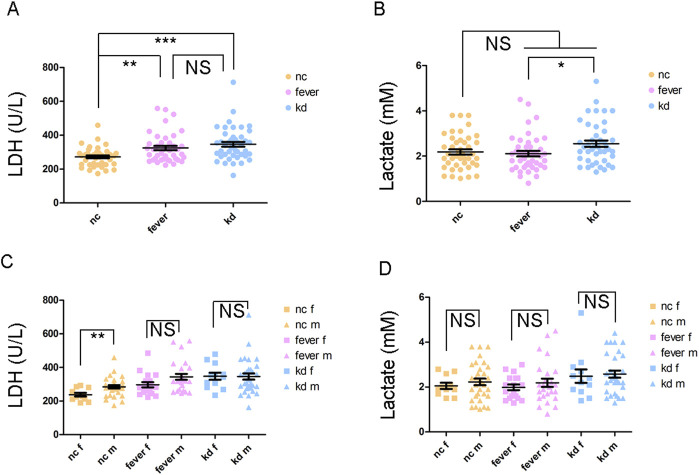
LDH and lactate levels in different groups of children. **(A)** LDH level of the children. **(B)** Lactate level of the children. **(C)** Comparison of LDH levels between female and male children. **(D)** Comparison of lactate levels between female and male children. “f” appended to “NC,” “Fever,” and “KD” denotes female, and “m” represents male; NS: not significant; *P < 0.05; **P < 0.01; ***P < 0.001.

A total of 44 children with fever, 44 children with KD, and 43 children with redundant prepuce or hernia (serving as normal controls, NC) were recruited. KD patients were enrolled according to the diagnostic criteria outlined in the American Heart Association’s 2017 KD guidelines (Newburger et al., 2004). Peripheral blood was collected to measure the levels of LDH and lactate in the serum (Figure 1A; Supplementary Table 1). This study adhered to the guidelines of the Declaration of Helsinki and was approved by the Ethics Committee of the Children’s Hospital of Soochow University (2022CS049). Written informed consent was obtained from the parents of the recruited children before they participated in the study. Lactate and LDH levels were measured using an automatic biochemical analysis system (AU5800, Beckman Coulter).

### Animals

All animal experiments were conducted in accordance with the Guide for the Care and Use of Laboratory Animals provided by the China National Institutes of Health and approved by the Animal Care and Use Committee of Soochow University (SUDA20220906A01). The mice were housed at the Children’s Hospital of Soochow University. Male C57BL/6 mice aged 3–4 weeks were randomly divided into two groups. The normal control (NC) group received intraperitoneal injections of phosphate-buffered saline (PBS), while the KD model group was injected with *Candida albicans* cell wall extracts. CAWS were administered intraperitoneally at a dose of 5 mg per day for 5 consecutive days, and the NC group received an equivalent volume of PBS via intraperitoneal injection. The CAWS used in this study were prepared from the *Candida albicans* strain NBRC1385 (Stock et al., 2016; Tada et al., 2008). On Days 14 or 28, after the final CAWS injection, the mice were anesthetized and sacrificed, and their heart tissues were harvested for subsequent examinations.

### Sample preparation and Western blotting of Kla proteins

Four hearts from mice in the PBS group on days 14 and 28 and four hearts from mice in the CAWS group on days 14 and 28 were collected. The hearts were minced and dissociated into single cells, following the instructions of Multi Tissue Dissociation Kit 2 (Miltenyi Biotec, 130-110-203). Red blood cells in the hearts were lysed using Red Blood Cell Lysis Buffer (Solarbio, R1010). After washing three times with precooled PBS, the cells were lysed with RIPA lysis buffer (Solarbio, R0010) supplemented with a protease inhibitor cocktail (Abcam, ab65621). The lactylation of proteins in the lysate was analyzed by Western blotting using an anti-L-lactyl lysine rabbit mAb antibody (PTMBio, PTM-1401RM) and HRP-conjugated goat anti-rabbit IgG (H + L) (Proteintech, SA00001-2).

### Sample preparation

Six hearts from mice in the PBS group and six hearts from mice in the CAWS group at Day 28 were obtained. Each group had three independent replicate samples, with two hearts combined into one sample. The samples were ground into cell powder using liquid nitrogen. Four volumes of lysis buffer (8 M urea and 1% protease inhibitor cocktail) were added, and the mixture was sonicated on ice for 3 min by using a high-intensity ultrasonic processor (Scientz). The lysate was centrifuged at 12,000 g for 10 min at 4°C, and the supernatant was collected. The protein concentration was determined using a BCA kit (Solarbio, PC0020), according to the manufacturer’s instructions. The sample was slowly added to the final concentration of 20% (m/v) trichloroacetic acid to precipitate the protein. The precipitate was collected by centrifugation at 4,500 g for 5 min at 4°C, washed three times with precooled acetone, and dried for 1 min. The sample was then redissolved in 200 mM TEAB and ultrasonically dispersed. Trypsin was added at a 1:50 trypsin-to-protein mass ratio for overnight digestion. The sample was reduced with 5 mM dithiothreitol for 30 min at 56°C and alkylated with 11 mM iodoacetamide for 15 min at room temperature in the dark. Finally, the peptides were desalted using a Strata X SPE Column.

### Proteomic analysis and lactylation proteomic analysis

For proteomic analysis, LC-MS/MS was performed. For lactylation proteomic analysis, lactylated peptides were enriched. Tryptic peptides were dissolved in NETN buffer (100 mM NaCl, 1 mM EDTA, 50 mM Tris-HCl, 0.5% NP-40, pH 8.0) and incubated overnight at 4°C with gentle shaking, using pre-washed anti-L-lactyl lysine antibody-conjugated agarose beads (PTM Bio, PTM-1404). The beads were washed four times with NETN buffer and twice with H_2_O. The bound peptides were eluted from the beads using 0.1% trifluoroacetic acid. The eluted fractions were combined and vacuum-dried. The resulting peptides were desalted using C18 ZipTip (Millipore), according to the manufacturer’s instructions. The tryptic peptides were dissolved in solvent A and directly loaded onto a home-made reverse-phase analytical column (25 cm length, 100 μm i. d.). The mobile phase consisted of solvent A (0.1% formic acid and 2% acetonitrile/in water) and solvent B (0.1% formic acid in acetonitrile). Peptides were separated using the following gradient: 0–18 min, 6%–22% B; 18–26 min, 22%–30% B; 26–27 min, 30%–80% B; and 27–30 min, 80% B, at a constant flow rate of 450 nL/min on a nanoElute UHPLC system (Bruker Daltonics). The peptides were subjected to analysis using a capillary source, followed by timsTOF HT mass spectrometry. The electrospray voltage applied was 1.6 kV. Precursors and fragments were analyzed at the TOF detector. The timsTOF Pro was operated in the data-independent parallel accumulation serial fragmentation (dia-PASEF) mode. The full MS scan was set as 100–1700 (MS/MS scan range), with MS/MS scans acquired in 8 PASEF (MS/MS mode) per cycle. The MS/MS scan range was set as 425–1025, and the isolation window was set as 25 m/z.

Building the spectral library: The DDA data were processed using Spectronaut (v.18) software, coupled with the Pulsar search engine. Tandem mass spectra were searched against Mus_musculus_10090_SP_20231220.fasta database (17,191 entries), concatenated with a reverse decoy database. The maximum number of missed cleavages was set to 2. Carbamidomethylation on Cys was specified as a fixed modification, while acetylation on the protein N-terminal, oxidation on Met, and lactylation were specified as variable modifications. The false discovery rate (FDR) of the protein, peptide, and PSM was adjusted to <1%. The corresponding spectral library was imported into Spectronaut (v.18) software to predict the retention time by nonlinear correction and then searched against the DIA data. Finally, LC-MS/MS analysis was carried out.

### Quantitative methods for proteomic analysis and lactylation proteomic analysis

In proteomic analysis, the raw LC-MS datasets were initially subjected to a database search. Subsequently, they were converted into matrices that included the normalized intensity of proteins. The normalized intensity refers to the raw intensity after the sample or batch effects have been corrected by use of the MSstats R package. After centralization, the normalized intensity (*I*) was transformed into the relative quantitative value (*R*). The formula is presented as follows, where *i* denotes the sample and *j* represents the protein:

$$Rij=Iij\;/\;Mean\left(Ij\right).$$

In lactylation proteomic analysis, the raw LC-MS datasets were first searched against a database. Subsequently, they were converted into matrices that encompass the intensities of peptides across samples. Next, the relative quantitative value of each Kla-modified peptide was calculated based on this intensity information through the following steps:

(1) The intensities of Kla-modified peptides (*I*) were centralized and then transformed into relative quantitative values (*R*). The formula is as follows, in which *i* represents the sample and *j* represents the modified peptide:
$$Rij=Iij\;/\;Mean\left(Ij\right).$$
(2) Since both proteomic analysis and Kla modification profiling were performed on the same sample cohort, the relative quantitative value of the modified peptide was divided by that of the corresponding protein. This step was performed to eliminate the influence of protein expression levels on Kla modification.

### Bioinformatics analysis

Gene Ontology (GO) analysis was performed using eggNOG-mapper software. GO IDs were extracted from the identified proteins based on the eggNOG database, and then the proteins were functionally classified and annotated according to cellular components (CCs), molecular functions (MFs), and biological processes (BPs). The Kyoto Encyclopedia of Genes and Genomes (KEGG) enrichment analysis was conducted based on the KEGG pathway database. Proteins were identified through BLAST comparison (BLASTP, e-value ≤ 1e^-4^). For each sequence, the annotation was based on the top-scoring comparison result.

### Statistical analysis

Data were analyzed using SPSS (version. 20). Continuous data were expressed as the mean ± standard error of the mean (S.E.M.). Regarding the LDH and lactate results, the Shapiro–Wilk test was employed to evaluate the normal distribution. When the data did not follow a normal distribution, the Mann–Whitney U-test was used to analyze the differences between groups. For the omics results and the normally distributed LDH and lactate results, the *t*-test was applied. A two-tailed *p-*value less than 0.05 was considered to indicate statistical significance.

In the omics analysis, proteins with a *p-*value less than 0.05 and a fold change greater than 1.5 were regarded as significantly upregulated proteins. Conversely, proteins with a *p-*value less than 0.05 and a fold change less than 0.667 were considered significantly downregulated proteins. Fisher’s exact test was used to assess the significance of the functional enrichment of differentially expressed proteins. Functional terms with a fold enrichment greater than 1.5 and a *p-*value less than 0.05 were deemed significant.

## Results

### The LDH and lactate concentrations were higher in KD patients

Peripheral blood was collected from the participants to measure the serum levels of LDH and lactate (Supplementary Table 1). The LDH level was significantly higher in both the fever and KD groups compared to the NC group (Figure 1A). However, there was no significant difference between the fever and KD groups (Figure 1A). Although the lactate level did not differ significantly between the fever and KD groups compared to the NC group, it was significantly higher in the KD group than in the fever group, with a fold change of 1.2 (Figure 1B). Given the higher incidence of KD in boys than in girls (Singh et al., 2015), the LDH and lactate levels in male and female KD patients were examined. No significant differences were observed (Figures 1C, D).

### Kla modification analysis of hearts from KD mouse model

**FIGURE 2:**
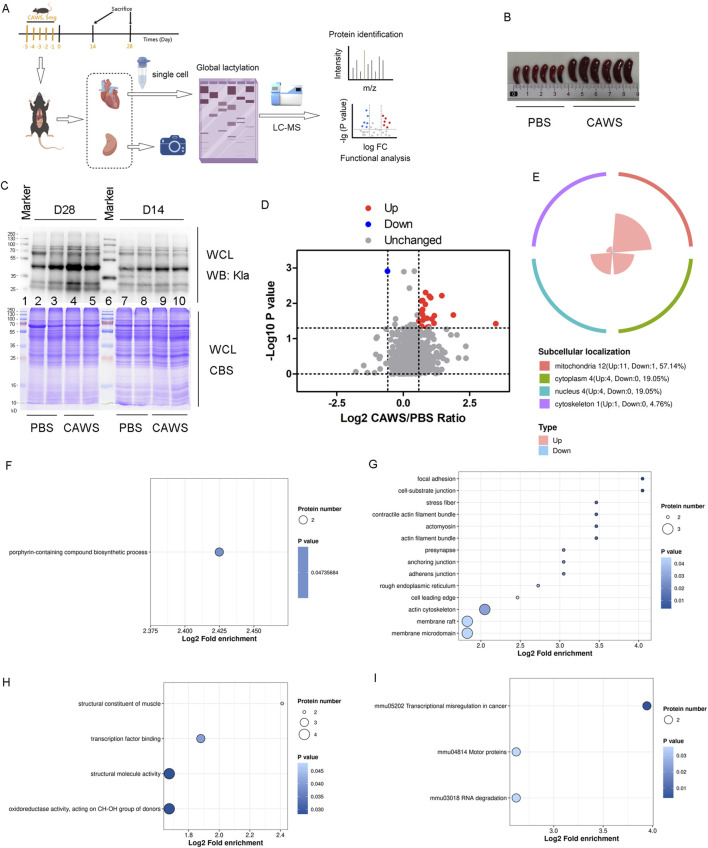
Kla modification in mouse hearts. **(A)** Schematic diagram of the mouse model experiments. **(B)** Spleens of mice treated with PBS or CAWS. **(C)** Kla modification levels in the hearts of PBS- and CAWS-treated mice on days 14 and 28. **(D)** Volcano plot of the altered Kla modification proteins. The FC value is greater than 1.5, and the *p*-value is less than 0.05. **(E)** Subcellular localization of altered Kla modification proteins. GO analysis encompassing **(F)** BPs, **(G)** CCs, and **(H)** MFs. **(I)** KEGG enrichment analysis of the Kla results. WCL, whole-cell lysate; CBT, Coomassie blue staining.

Previous results showed elevated lactate levels in KD. Since lactate is associated with lactylation, which is crucial in cardiovascular and inflammatory diseases (Zhu et al., 2024; You et al., 2024), the Kla levels in KD hearts were investigated. A schematic illustration of the mouse model experiments was provided (Figure 2A). The KD models were confirmed by splenomegaly (Figure 2B). Two hearts from KD mouse models on days 14 and 28 were collected, and Kla modification in the hearts was examined (Figure 2C). The alteration was more pronounced on Day 28 than on Day 14. Therefore, six KD mouse models and six normal controls were selected on Day 28. Hearts from two mice in each group were pooled and considered one sample. It was identified that Kla modification at 23 sites in 20 proteins [Atp5if1, Myl3, Idh2, Actb, and Hspd1 (two sites), H3c1 (two sites), H3c2 (two sites), Csrp1, Vdac3, Ndufa12, Bdh1, Me3, Hadha, Pnpt1, Oxnad1, Mrpl37, Cox6c, Ndufa6, Uqcrb, and Acot9] was significantly upregulated, and at one site in one protein (Fech), it was significantly downregulated (Figure 2D; Supplementary Table 2). Among these proteins, the majority (57.14%) were located in the mitochondria (Figure 2E). The GO analysis, including BPs (Figure 2F), CCs (Figure 2G), and MFs (Figure 2H) and the Kyoto Encyclopedia of Genes and Genomes (KEGG) enrichment analysis (Figure 2I) were performed. The enrichment of the “structural constituent of muscle” pathway in the MF term suggested an association between lactylation and the abnormal cardiac muscle structure affected by KD.

### Proteomic analysis of hearts in KD mouse models

**FIGURE 3:**
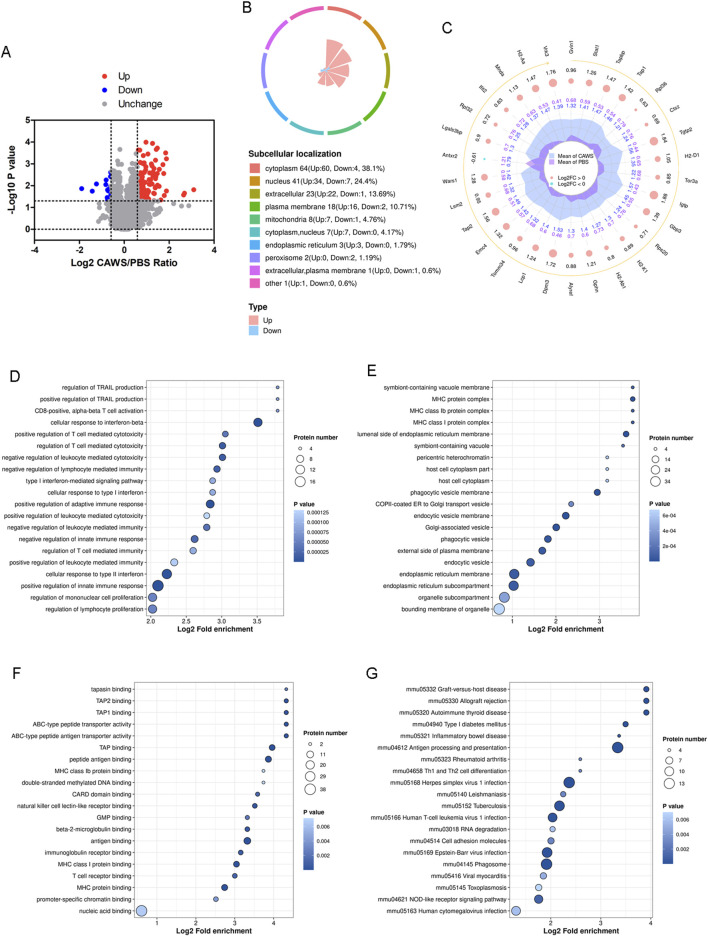
Proteomic analysis of the mouse hearts. **(A)** Volcano plot of the altered expression proteins. The FC value is greater than 1.5, and the *p*-value is less than 0.05. **(B)** Subcellular localization of the altered proteins. **(C)** Top 30 altered proteins. GO analysis encompassing **(D)** BPs, **(E)** CCs, and **(F)** MFs. **(G)** KEGG enrichment analysis of the proteomic results.

In this investigation, proteomic analysis was carried out using protein samples identical to those employed in the lactylome analysis (Supplementary Figure 1). The analysis identified 150 upregulated proteins and 18 downregulated proteins (Figure 3A; Supplementary Table 3). Significantly, 38.1% of these differentially expressed proteins were located in the cytoplasm (Figure 3B). The top 30 differentially expressed proteins (Gvin1, Stat1, Tapbp, Tap1, Rpl36, Ctsz, Tgtp2, H2-D1, Tor3a, Igtp, Gbp3, Rps20, H2-K1, H2-Ab1, Gphn, Alyref, Dpm3, Lcp1, Tomm34, Emc4, Tap2, Lsm2, Wars1, Antxr2, Lgals3bp, Rpl32, Ifit2, Mnda, H2-Aa, and Vrk3) were shown (Figure 3C). Subsequently, GO analysis was performed, encompassing BPs (Figure 3D), CCs (Figure 3E), and MFs (Figure 3F). Concurrently, the Kyoto Encyclopedia of Genes and Genomes (KEGG) enrichment analysis was conducted (Figure 3G). The results demonstrated an enrichment of pathways related to immunity. For instance, the “regulation of lymphocyte proliferation” (Figure 3D), “MHC protein complex” (Figure 3E), “T cell receptor binding” (Figure 3F), and “Th1 and Th2 cell differentiation” (Figure 3G) were among the enriched pathways. This suggests that lymphocytes and T cells play important roles in the development of KD.

### Alterations in Kla modification after normalization

**FIGURE 4:**
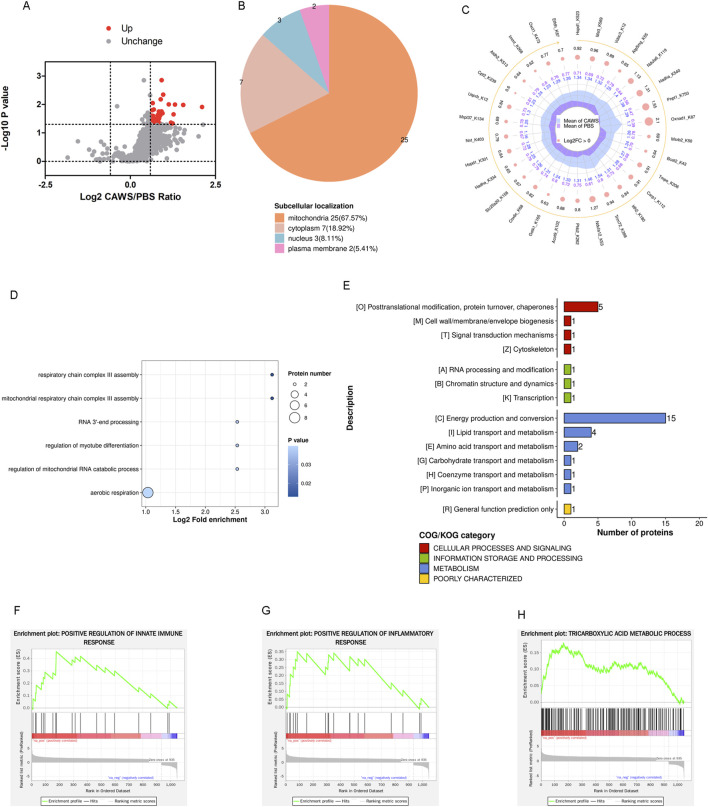
Normalized Kla modification in mouse hearts. **(A)** Volcano plot of the normalized altered Kla-modified proteins. The FC value is greater than 1.5, and the *p*-value is less than 0.05. **(B)** Subcellular localization of the normalized altered Kla-modified proteins. **(C)** Top 30 altered Kla-modified proteins. **(D)** BP term of GO analysis. **(E)** COG classification of Kla-modified proteins. The GSEA indicated the pathways of **(F)** positive regulation of innate immune responses, **(G)** positive regulation of inflammatory responses, and **(H)** tricarboxylic acid metabolic processes were affected.

The Kla modification data were normalized to the proteomic results. Through this normalization process, it was discovered that 41 sites on 37 proteins [Phb2, Bcat2, Cox4i1, Hspa9, Aldh2, Acadvl, Cpt2, Idh2, Hspd1 (2 sites), Csrp1, Atp5pf, Trim72, Ogdh, Vdac3 (2 sites), Tmpo, Nnt, Ckmt2, Lrpprc, Msrb2, Ndufa12, Me3, Hadha (2 sites), Immt, Pnpt1, Oxnad1, Etfdh, Mrpl37, Macrod1, Aco2, Cox6c, Atp5mg (2 sites), Ndufa6, Oxct1, Uqcrb, Gstk1, Acot9, and Slc25a20] were upregulated. Notably, no downregulated sites were detected (Figure 4A). Approximately 67.57% of these altered proteins were localized in the mitochondria (Figure 4B). The top 30 sites of proteins [Hspd1 (2 sites), Me3, Vdac3, Atp5mg, Ndufa6, Hadha, Pnpt1, Oxnad1, Msrb2, Bcat2, Tmpo, Csrp1, Idh2, Trim72, Ndufa12, Phb2, Acot9, Gstk1, Cox6c, Slc25a20, Hadha, Nnt, Mrpl37, Uqcrb, Cpt2, Aldh2, Immt, Oxct1, and Etfdh] were shown (Figure 4C). The BP term from the analysis suggested that aerobic respiration was disrupted (Figure 4D). The Clusters of Orthologous Groups (COG) classification of the lactylated proteins indicated an alteration in energy production and conversion (Figure 4E). Additionally, gene set enrichment analysis (GSEA) revealed that pathways involved in the positive regulation of innate immune responses (Figure 4F), positive regulation of inflammatory responses (Figure 4G), and tricarboxylic acid metabolic processes (Figure 4H) were significantly affected. This indicates that Kla modification may play a crucial role in modulating these key biological processes during the development of Kawasaki disease.

## Discussion

In this study, we pioneered the exploration of Kla modification in KD. Our findings demonstrated associations between both lactate levels and Kla modification and KD. Moreover, inflammatory response-related pathways were significantly enriched in both the proteomic and lactylome analyses.

Physical activity typically increases lactate production in muscles. However, children with fever or those in the acute phase of KD may decrease their physical activity due to discomfort. Therefore, excluding the impact of physical activity, the elevated lactate level in KD patients is likely attributed to the Warburg effect during inflammation. This effect stimulates cells to enhance glycolysis, resulting in increased lactate production (Bögel et al., 2024; Hu et al., 2024). In this study, we observed that the lactate level was significantly higher in KD patients (Figure 1B). This finding initially suggested that lactate levels could potentially serve as a biomarker for differentiating KD from other febrile diseases.

However, this conclusion was merely derived from theoretical statistical analysis. In practical clinical settings, it is impracticable to diagnose KD in a patient solely based on lactate levels. The data within each group exhibit substantial variability, and the fold change in lactate levels between the KD and fever groups is merely 1.2. The significantly higher lactate levels in KD patients went unnoticed until statistical analysis was conducted. When examining the original lactate data (Supplementary Table 1), it is clear that distinguishing KD from other fevers based on lactate levels is not feasible.

KD is a medium-vessel vasculitis, and it likely recruits or activates different subsets of immunocytes compared to typical fevers. Consequently, glycolysis may be enhanced in KD, leading to increased lactate production. However, the differences in immune responses between KD and fever remain to be investigated. Additionally, early biomarkers capable of differentiating KD from other febrile diseases are yet to be identified.

Furthermore, we found no significant difference in lactate or LDH levels between male and female KD patients. This suggests that the higher susceptibility to KD in boys is unlikely to be attributed to lactate or LDH levels (Figures 1C, D). We also noticed that LDH and lactate levels do not always correlate. In the fever group, the LDH levels increased significantly, while the lactate levels did not. This may be because LDH plays a reversible role in the conversion between pyruvic acid and lactate. The distinct role of LDH might be associated with the disease outcome, whether it progresses to KD or remains a simple fever.

Proteomic analysis indicated that the differentially expressed proteins were associated with multiple pathways linked to immune cells and immune responses (Figure 3D). We observed that leukocytes, particularly CD8^+^ T cells, were still involved in the subacute phase of KD. Given that CD8^+^ T cells eliminate target cells by secreting cytotoxic molecules and pro-inflammatory cytokines (Zandhuis et al., 2024), they may contribute to the development of coronary artery lesions (CALs). The functions of CD8^+^ T cells in this context have also been corroborated by other studies (Rowley and Shulman, 2018).

Most of the altered proteins identified through proteomic analysis were localized in the cytoplasm and nucleus. Notably, only 4.76% of these proteins were found in the mitochondria (Figure 3B). In contrast, the majority of lactylated proteins were localized within the mitochondria (Figures 2E, 4B). It is well-established that mitochondria are strongly associated with KD (An et al., 2023; Lin et al., 2023). Although an increase in mitochondrial protein lactylation has been reported in acetaminophen-induced liver injury (Hong et al., 2024), the significance of lactylation in mitochondria remains unclear.

Since intratumoral lactate could be transported into macrophages to support the tricarboxylic acid (TCA) cycle (Fang et al., 2023), we hypothesized that during the progression of KD, increased lactate might be shuttled into the mitochondria to fuel the TCA cycle. This, in turn, could lead to an elevation in mitochondrial protein lactylation. However, the functions of protein lactylation in the mitochondria still need to be further investigated in future studies.

In summary, this study investigated the expression of proteins and their Kla modification in the hearts of KD mouse models. This research enhanced our understanding of the role of Kla modification in the development of KD. Moreover, it holds the potential to guide the development of targeted therapies for the prevention of KD or for achieving a better prognosis.

## Data Availability

The datasets presented in this study can be found in online repositories. The names of the repository/repositories and accession number(s) can be found in the article/Supplementary Material.

## Supplementary material

SUPPLEMENTARY FIGURE 1 Schematic representation of the pooled samples subjected to proteome and Kla modification analysis.
