# Supplementary figures and images for: Lysine lactylation analysis of proteins in the heart of the Kawasaki disease mouse model
Source: Front Cell Dev Biol. 2025 Mar 6;13:1550220. doi: 10.3389/fcell.2025.1550220 (PMC11922914; doi:10.3389/fcell.2025.1550220)

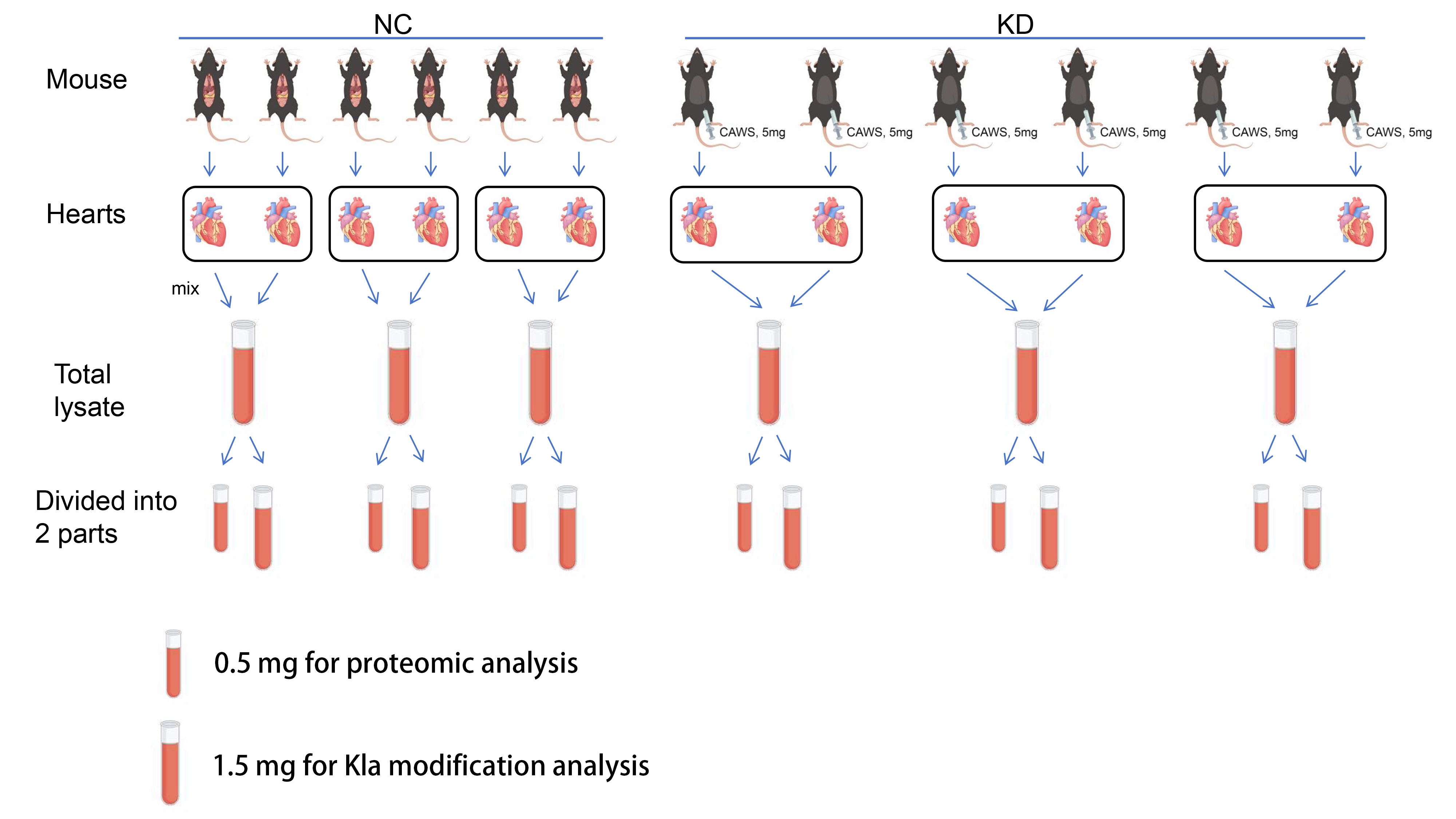

Supplement: Supplementary file 3 [file Image1.tif]
